# Dead Crow Reports and Location of Human West Nile Virus Cases, Chicago, 2002

**DOI:** 10.3201/eid1005.030603

**Published:** 2004-05

**Authors:** John T. Watson, Roderick C. Jones, Kevin Gibbs, William Paul

**Affiliations:** * Centers for Disease Control and Prevention, Atlanta, Georgia, USA; † Chicago Department of Public Health, Chicago, Illinois, USA

**Keywords:** West Nile virus, epidemiology, disease outbreaks, population surveillance, geographic information systems

## Abstract

During the summer and fall of 2002, an epidemic (223 cases) and epizootic of West Nile virus infections occurred in Chicago. Retrospective spatial analysis demonstrated that age-adjusted human case rates were three times higher inside geographic areas with high early-season crow deaths than outside these areas.

West Nile virus (WNV) activity was first identified in North America in New York City in 1999 (*1*). In each subsequent year, the WNV epidemic and epizootic have spread across the continent (*2*), as new susceptible human, avian, and equine populations are exposed to the virus.

During previous seasons of WNV transmission in most affected areas of the United States, the infection and deaths of crows (*Corvus brachyrynchos*) have preceded the detection of human infections. Tracking sightings of dead crows is advocated as an integral aspect of WNV activity surveillance systems (*3*,*4*). The utility of dead crow surveillance in predicting the intensity or geographic extent of subsequent human epidemics, however, is uncertain (*5*).

During the summer of 2002, an epizootic and epidemic of WNV infections occurred in Chicago, a city of 2.9 million residents and an area of 231 square miles. We conducted a retrospective study to assess the spatial relationship between the locations of dead crow sightings reported early in the transmission season and the residences of persons subsequently reported to have cases of WNV infection.

## The Study

Human cases of aseptic meningitis and encephalitis are reportable conditions in Illinois. During 2002, the Illinois Department of Public Health determined WNV-positive cases by using its own laboratory as well as outside laboratory services. Upon notification by the state health department of a WNV-positive result in a Chicago resident, the Chicago Department of Public Health conducted an epidemiologic investigation of the case.

Human cases of WNV infection were classified as WNV meningoencephalitis or WNV fever. Cases in patients with acute onset of signs and symptoms consistent with meningitis or encephalitis were defined as WNV meningoencephalitis. Cases without acute onset of signs and symptoms consistent with meningitis or encephalitis were classified as WNV fever.

In Chicago, citizens may call the city’s nonemergency hotline to report dead bird sightings or to request pickup and disposal of a dead bird. For each such call, operators record the date of the call, the type of bird reported, and the dead bird’s street location.

**Figure 1:**
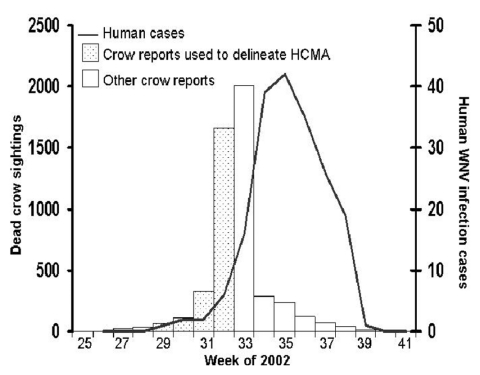
Dead crow sightings reported to the Chicago nonemergency hotline, by week of report, and human cases of West Nile virus infection, by week of illness onset, Chicago, June 16 (beginning of week 25) through October 12 (end of week 41), 2002. HCMA, high crow-mortality area.

The first serologic confirmation of human WNV infection in a Chicago resident was reported on August 12, 2002. By the end of 2002, a total of 223 cases of WNV infection were reported in Chicago residents, with onsets of illness occurring from July 18 to October 7. The human epidemic peaked during the week ending August 31 (week 35). The first dead crow sighting recorded by the nonemergency hotline occurred on July 1; by October 19, a total of 3,837 dead crow sightings at distinct addresses had been reported through the hotline. These reports peaked during the week ending August 17 (week 33) (Figure 1).

Spatial analysis was used to assess the relationship between the locations of dead crow sightings reported before the first human case of WNV infection was detected and the reported residence of human WNV case-patients. Spatial analysis was conducted by using ArcView Spatial Analyst version 1.1 in ArcView version 3.2, and maps were generated with ArcView version 8.2 (all software: Environmental Systems Research Institute, Inc., Redlands, CA). Dead crow sightings reported through the hotline from July 1 through August 11 were geocoded and mapped. Smoothed dead crow densities were generated by using kernel estimation to calculate density and a bandwidth radius of 1 mile. The kernel function was used to describe mathematically the geographic density of crow deaths by assigning a value to a point on the map; the value is a function of that point’s proximity to surrounding crow deaths (*6*). The dead crow density values were reclassified into deciles. All map areas with a density value classified in the top nine deciles were delineated and designated as high crow-mortality areas. Such areas were then expanded to conform to census tract boundaries.

Human cases of WNV infection were geocoded and mapped by using the case-patient’s reported street address of residence. Human case rates were calculated on the basis of the 2000 census population within and outside the high crow-mortality areas. To control for age, the data were stratified by age group (0–24 years, 25–49 years, 50–64 years, >65 years); effect modification and confounding were assessed; and the rates within and outside the high crow-mortality areas were compared by calculating a Mantel-Haenszel weighted incidence ratio with Greenland-Robins 95% confidence intervals (CI) in EpiInfo version 6.04 (Centers for Disease Control and Prevention, Atlanta, GA). Because WNV fever case-patients were often seen as outpatients and their inclusion could have introduced diagnostic access bias into the findings, the analysis was conducted a second time, with patients limited to those meeting the WNV meningoencephalitis case classification.

**Figure 2:**
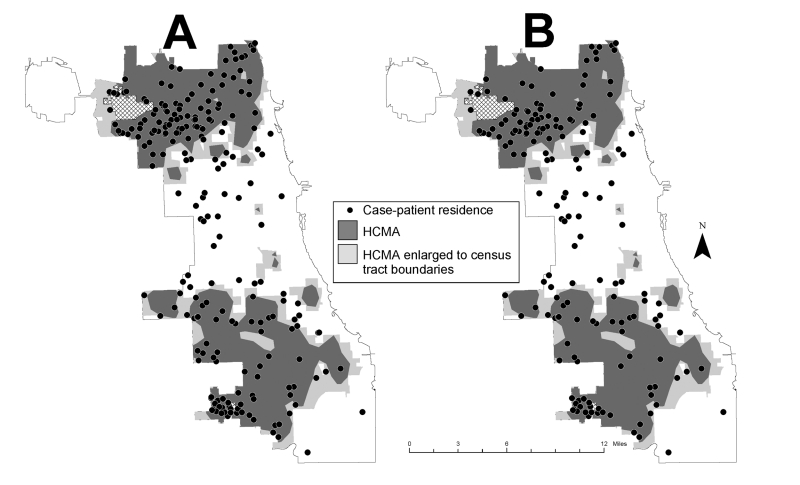
Chicago map with high crow-mortality areas (HCMAs) and reported residences of A) West Nile virus (WNV)-infected case-patients, or B) WNV meningoencephalitis case-patients (WNV fever cases excluded), 2002.

Of 3,837 dead crow sightings reported through week 32, a total of 3,833 (>99%) had address information sufficient for geocoding. Home address data were obtained for 219 (98%) of the 223 Chicago case-patients. The high crow-mortality area covered 124 square miles (54% of Chicago’s surface area), and 179 (82%) of the geocoded case-patients resided within it’s the city’s boundaries (Figure 2A). The crude case rate for WNV infection was 10.8/100,000 inside the high crow-mortality areas compared with 3.2/100,000 outside (Mantel-Haenszel weighted incidence ratio 3.0, 95% CI 2.1 to 4.2). For WNV meningoencephalitis only (n = 166), the crude case rate was 7.8/100,000 inside the high crow-mortality areas compared to 2.9/100,000 outside (Figure 2B) (Mantel-Haenszel weighted incidence ratio 2.3, 95% CI 1.6 to 3.4). The spatial association was not confounded by age group, nor did the strength of association differ significantly by age group.

## Conclusions

We identified a spatial association between early-season crow deaths and residences of WNV-infected case-patients in Chicago. The findings indicate that a functioning dead-crow surveillance system could assist in prioritizing geographic areas for mosquito-control interventions, such as risk communication and larval and adult mosquito control. These interventions could occur before, or concurrently with, the identification of WNV-infected case patients in a given transmission season.

The success of dead crow surveillance systems depends in part upon public participation, and the Chicago nonemergency hotline—a simple, well-recognized, and well-publicized means of collecting dead crow data—was an essential prerequisite to this analysis. One potential source of bias that accompanies these data, nonetheless, is the self-selection of persons who report crow deaths. Residents of certain neighborhoods might be more inclined to notify their local government upon seeing a dead bird than those in other neighborhoods.

WNV-associated avian deaths have historically preceded human illness, and in Chicago, the epizootic peaked approximately 2 weeks before the human epidemic. Real-time analysis of incoming dead crow reports might be especially important because of the time lag between illness onset, confirmatory testing for WNV infection, report to the local health authority, and initiation of an epidemiologic case investigation to identify and adequately describe WNV-related human illness. In Chicago in 2002, the first case of WNV infection in a resident was reported on August 12, 13 days after the patient’s onset of illness.

Whether a similar spatial association between early-season crow deaths and residences of WNV-infected case-patients will be evident in future seasons is unknown, as an estimated 81% of the Chicago-area crow population is thought to have died in 2002 (*7*). Nonetheless, our experience highlights the potential utility of spatial analysis of dead crow reports in prioritizing geographic areas for mosquito-control interventions.
